# Supplementary material for: Dynamic transcriptomic profiles of zebrafish gills in response to zinc supplementation
Source: BMC Genomics. 2010 Oct 11;11:553. doi: 10.1186/1471-2164-11-553 (PMC3091702; doi:10.1186/1471-2164-11-553)
Supplement: Additional file 2 — Interactive Direct Interaction Network representing the molecular interactions between zinc, copper, iron, calcium and proteins encoded by transcripts changed by zinc supplementation. Mini web-site containing index.html and hyperlinked pages in subdirectory describing a Direct Interaction Network automatically generated based on curated interactions contained within the proprietary PathwayArchitect database. Ovals represent proteins and the circles symbolize metal ions. Objects are coloured by their abundance in zebrafish at the time-point they were significantly different from the control is a scale from -4 fold (dark green) to +4 fold (dark red). Where significant differences were found at more than one time-point, the colour overlay shows expression at the first instance. Dark blue squares denote 'binding', and light blue squares 'expression'; green squares stand for 'regulation', green diamonds for 'metabolism', and green circles for 'promoter binding'. Arrow heads indicate directionality of the interaction where annotated. All nodes and edges can be further interrogated by selecting the relative area of the image. [file 1471-2164-11-553-S2.zip › PathwayArchitect Zn xs DIN/541272.html]

# EXPRESSION:

|  |  |
| --- | --- |
| Type | EXPRESSION |
| Effect | None |


---

|  |  |
| --- | --- |
| Score | 0 |


---

|  |  |
| --- | --- |
| Reference Count | 17 |


---

|  |  |
| --- | --- |
| Mechanism | Unknown |


---

|  |  |
| --- | --- |
| Reference:0 || Sentence | "These results suggest that induction of the MMP-13 gene by TNF-alpha is mediated by ERK, p38, and JNK MAP kinases as well as AP-1 and NF-kappaB transcription factors." |
| PMID | 12878172 |
| Year | 2003 |
| Species | Human |
| Journal | Exp Cell Res |
| RefScore | 0 |
| Source | PArchNLP |
  |
|  |  |


---

|  |  |
| --- | --- |
 Reference:1 || Sentence | "ETS-1 also markedly potentiated enhancement of MMP-1 promoter by both c-Jun and JunB, whereas ERGB/Fli-1 augmented only the effect of c-Jun." |
| PMID | 9178763 |
| Year | 1997 |
| Species | Mouse |
| Journal | Oncogene |
| RefScore | 1 |
| Source | PArchNLP |
  ||  |  |


---

|  |  |
| --- | --- |
 Reference:2 || Sentence | "Interestingly, PU.1 abolished induction of MMP-1 promoter by both c-Jun and JunB." |
| PMID | 9178763 |
| Year | 1997 |
| Species | Mouse |
| Journal | Oncogene |
| RefScore | 0 |
| Source | PArchNLP |
  ||  |  |


---

|  |  |
| --- | --- |
 Reference:3 || Sentence | "Adenovirus-mediated expression of dominant negative p38alpha and c-Jun potently inhibited induction of MMP-13 expression in gingival fibroblasts by TGF-beta1." |
| PMID | 10601295 |
| Year | 1999 |
| Species | Human |
| Journal | J Biol Chem |
| RefScore | 0 |
| Source | PArchNLP |
  ||  |  |


---

|  |  |
| --- | --- |
 Reference:4 || Sentence | "In three out of four lines studied, the AP-1-dependent collagenase gene was expressed at a low level." |
| PMID | 8479751 |
| Year | 1993 |
| Species | Rat |
| Journal | Oncogene |
| RefScore | 1 |
| Source | PArchNLP |
  ||  |  |


---

|  |  |
| --- | --- |
 Reference:5 || Sentence | "To determine whether an increase in jun-B at the same time as c-fos and c-jun would inhibit collagenase gene transcription, we cotransfected an expression vector for jun-B with a rat collagenase promoter-reporter gene construct." |
| PMID | 9192514 |
| Year | 1997 |
| Species | Rat |
| Journal | Calcif Tissue Int |
| RefScore | 2 |
| Source | PArchNLP |
  ||  |  |


---

|  |  |
| --- | --- |
 Reference:6 || Sentence | "Both interaction of Cbfa/Runt proteins with AP-1 and the presence of a functional proximal OSE2 site are required for enhanced transcriptional activity of the mmp13 promoter in transient transfected fibroblasts and in PTH-treated osteosarcoma cells." |
| PMID | 11274169 |
| Year | 2001 |
| Species | Mouse |
|  | Human |
| Journal | J Biol Chem |
| RefScore | 2 |
| Source | PArchNLP |
  ||  |  |


---

|  |  |
| --- | --- |
 Reference:7 || Sentence | "Over-expression of c-jun leads to trans-activation of the collagenase promoter in both cell types, suggesting that c-jun is a ubiquitous inducer of collagenase gene expression." |
| PMID | 8631909 |
| Year | 1996 |
| Species | Mouse |
|  | Human |
| Journal | J Biol Chem |
| RefScore | 2 |
| Source | PArchNLP |
  ||  |  |


---

|  |  |
| --- | --- |
 Reference:8 || Sentence | "These results show that three structurally distinct ETS transcription factors differently modulate AP-1 dependent upregulation of MMP-1 gene expression." |
| PMID | 9178763 |
| Year | 1997 |
| Species | Mouse |
| Journal | Oncogene |
| RefScore | 1 |
| Source | PArchNLP |
  ||  |  |


---

|  |  |
| --- | --- |
 Reference:9 || Sentence | "Transient transfection of BAL-17 B cells with reporter gene constructs showed that B cell AP-1 failed to trans-activate the TRE-containing human collagenase gene promoter, for which activity is dependent upon functional expression of cellular c-Jun." |
| PMID | 1634770 |
| Year | 1992 |
| Species | Mouse |
| Journal | J Immunol |
| RefScore | 1 |
| Source | PArchNLP |
  ||  |  |


---

|  |  |
| --- | --- |
 Reference:10 || Sentence | "These data indicate that, in contrast to most LPS effects, AP-1, but not nuclear factor-kappaB, mediates LPS induction of collagenase transcription in macrophagelike cells." |
| PMID | 8621773 |
| Year | 1996 |
| Species | Mouse |
|  | Human |
| Journal | J Clin Invest |
| RefScore | 1 |
| Source | PArchNLP |
  ||  |  |


---

|  |  |
| --- | --- |
 Reference:11 || Sentence | "Mutation of the first AP-1 site resulted in major reduction of the basal level of the MMP-1 promoter activity, supporting the notion that the AP-1 consensus sequence is essential for the constitutive expression of the MMP-1 gene." |
| PMID | 9266691 |
| Year | 1997 |
| Species | Mouse |
|  | Human |
| Journal | Eur J Biochem |
| RefScore | 1 |
| Source | PArchNLP |
  ||  |  |


---

|  |  |
| --- | --- |
 Reference:12 || Sentence | "Previous studies showed that palytoxin simulates an ERK-dependent selective increase in the c-Fos content of AP-1 complexes that bind to the promoter of the MMP-13 gene." |
| PMID | 14575638 |
| Year | 2003 |
| Species | Mouse |
| Journal | Toxicol Appl Pharmacol |
| RefScore | 1 |
| Source | PArchNLP |
  ||  |  |


---

|  |  |
| --- | --- |
 Reference:13 || Sentence | "Functional analysis of the collagenase-3 promoter showed that both the activator protein-1 (AP-1) site and the runt domain (RD) binding site were required for maximal constitutive expression of collagenase-3 in MDA cells." |
| PMID | 10891395 |
| Year | 2000 |
| Species | Human |
| Journal | Mol Cell Biol Res Commun |
| RefScore | 1 |
| Source | PArchNLP |
  ||  |  |


---

|  |  |
| --- | --- |
 Reference:14 || Sentence | "In conclusion, bFGF increases collagenase-3 gene transcription, an effect mediated through an AP-1 site, due to the induction or activation of Jun and Fos family transcription factors." |
| PMID | 10830307 |
| Year | 2000 |
| Species | Human |
|  | Rat |
| Journal | Endocrinology |
| RefScore | 0 |
| Source | PArchNLP |
  ||  |  |


---

|  |  |
| --- | --- |
 Reference:15 || Sentence | "We demonstrate that c-Jun and Jun-B nevertheless differ markedly in their ability to activate AP-1 responsive genes. c-Jun is an efficient activator of the c-jun and collagenase promoters, which contain a single TRE; Jun-B is not." |
| PMID | 2513128 |
| Year | 1989 |
| Species | Human |
|  | Mouse |
| Journal | Cell |
| RefScore | 3 |
| Source | PArchNLP |
  ||  |  |


---

|  |  |
| --- | --- |
 Reference:16 || Sentence | The activator protein-1 (AP-1) and runt domain binding (Runx/RD/Cbfa) sites and their respective binding proteins, c-Fos/c-Jun and Runx2 (Cbfa1), regulate the rat matrix metalloproteinase-13 (MMP-13) promoter in both parathyroid hormone (PTH)-treated and differentiating osteoblastic cells in culture. |
| PMID | 16639721 |
| Year | 2006 |
| Species | Mouse |
|  | Rat |
| Journal | J Cell Biochem |
| RefScore | 1 |
| Source | PArchNLP |
  |


---

|  |  |
| --- | --- |
